# Breast Acoustic Parameter Reconstruction Method Based on Capacitive Micromachined Ultrasonic Transducer Array

**DOI:** 10.3390/mi12080963

**Published:** 2021-08-14

**Authors:** Yu Pei, Guojun Zhang, Yu Zhang, Wendong Zhang

**Affiliations:** State Key Laboratory of Dynamic Testing Technology, North University of China, Taiyuan 030051, China; 15513096651@163.com (Y.P.); 15198201976@163.com (Y.Z.); wdzhang@nuc.edu.cn (W.Z.)

**Keywords:** USCT, CMUT array, sound speed, acoustic attenuation, reconstruction method

## Abstract

Ultrasound computed tomography (USCT) systems based on capacitive micromachined ultrasonic transducer (CMUT) arrays have a wide range of application prospects. For this paper, a high-precision image reconstruction method based on the propagation path of ultrasound in breast tissue are designed for the CMUT ring array; that is, time-reversal algorithms and FBP algorithms are respectively used to reconstruct sound speed distribution and acoustic attenuation distribution. The feasibility of this reconstruction method is verified by numerical simulation and breast model experiments. According to reconstruction results, sound speed distribution reconstruction deviation can be reduced by 53.15% through a time-reversal algorithm based on wave propagation theory. The attenuation coefficient distribution reconstruction deviation can be reduced by 61.53% through FBP based on ray propagation theory. The research results in this paper will provide key technological support for a new generation of ultrasound computed tomography systems.

## 1. Introduction

High-sensitivity transducer arrays and high-precision acoustic parameter reconstruction methods are the key factors of high-performance USCT systems. In terms of ultrasonic transducers, piezoelectric transducers are most commonly used in the USCT system. However, the image resolution is limited by the consistency of transducers. CMUT fabricated by MEMS micromachining technology has the advantages of broadband, high sensitivity, low power consumption, low cost, high consistency, and high integration, which can meet the needs of developing a USCT system with higher resolution [1]. In the new generation of ultrasound imaging systems, a USCT system based on a CMUT array has a wide range of application prospects.

In terms of the acoustic parameter reconstruction method, USCT uses both transmission and reflection data to provide quantitative images of breast tissue acoustic properties [2,3,4,5,6]. Sound speed is the most studied propagation characteristic since it has a strong correlation with tissue density [7]. Sound speed images can provide more details of different breast tissues shapes and edges, which is helpful in distinguishing benign and malignant structures [8,9]. On the other hand, compared with sound speed, acoustic attenuation varies more with tissue types, which can provide enhanced contrast for different types of tissues [5]. Therefore, it can significantly improve the detection rate of breast micro-lesions. In conclusion, the combination of sound speed images and acoustic attenuation images can better distinguish benign and malignant tumors. According to now available literature, typical USCT systems often use a tomography algorithm based on ray theory to inverse the sound speed of breast tissue [10,11,12,13]. However, the solution of ray acoustics is not the exact one to the wave equation but rather the approximate solution under the high-frequency condition. The following assumptions must be met when it is used: first, the frequency of the acoustic wave must be high enough, and wave length should be far smaller than the size of acoustic scatter; second, there should be no energy loss in the process of sound propagation; and third, the parameters of the medium should not change with time. In fact, the range of ultrasound used by the existing breast USCT system is mainly concentrated in 1.5 MHz–3.5 MHz [14,15,16,17,18,19,20,21,22,23,24,25] frequencies, which are not high-frequency ultrasound signals. The propagation distance of ultrasound signals is concentrated in 15 cm–20 cm, in which the energy of ultrasound signals is lost. This is also what should be considered when reconstructing images. In addition, frequency and propagation distances of ultrasound signals are mutually restricted [26]. Take 3 MHz as an example: its wavelength is about 0.5 mm. The size of an early breast cancer lesion is relatively small. When it is less than the wavelength, the ultrasound produces diffraction and scattering and the part that propagates along a straight line is very small [27]. Therefore, a transmission signal carries little information about lesion tissue; furthermore, the only information needed for reconstruction of sound speed image is an estimation of the transition time. At present, the correlation function method is the most accurate and the most commonly used to extract transition time. However, this method cannot deal with the coupling signal in the received signal, the nonlinearity of circuit, or the waveform distortion. The aforementioned reasons lead to the low resolution of reconstructed sound speed image based on ray theory, and it is not possible to accurately reconstruct the a tiny lesion’s tissue for which the size is smaller than the ultrasound wavelength. To this point, ultrasound tomography based on the exact solution to the wave equation is the most accurate reconstruction method of sound speed image and has been studied by different authors. The most famous are seismology and medical imaging [28,29], but sound speed reconstruction based on wave theory is usually performed in the time domain, ignoring the existence of acoustic attenuation [30,31,32,33]. In recent years, it has been reported that the effect of acoustic attenuation reconstruction using wave theory in the frequency domain is not very good [34].

In view of the above problems in the reconstruction of breast tissue acoustic parameters, this paper proposes a reconstruction method based on ultrasound propagation characteristics in breast tissue oriented to the CMUT ring array. The time-reversal algorithm and FBP algorithm, respectively, are used to reconstruct the sound speed image and the acoustic attenuation image. The projection data required by the two methods can be obtained from the same received signals, which not only ensures the accuracy of image reconstruction but also reduces the calculation time. This method is possible to accelerate the clinical application of breast USCT systems. In the following contents, the structure of CMUT and ultrasonic tomography systems based on the CMUT ring array are introduced initially. Subsequently, taking a CMUT ring array as an example, a reconstruction algorithm of sound speed and acoustic attenuation is given, and an actual implementation method is proposed under the background of USCT. Lastly, numerical experiments and breast model experiments are implemented to verify the accuracy and feasibility of using wave theory to reconstruct sound speed and ray theory to reconstruct acoustic attenuation. The research results in this paper lay a good theoretical and practical foundation for the new generation of USCT systems.

## 2. Model Creation

**Figure 1:**
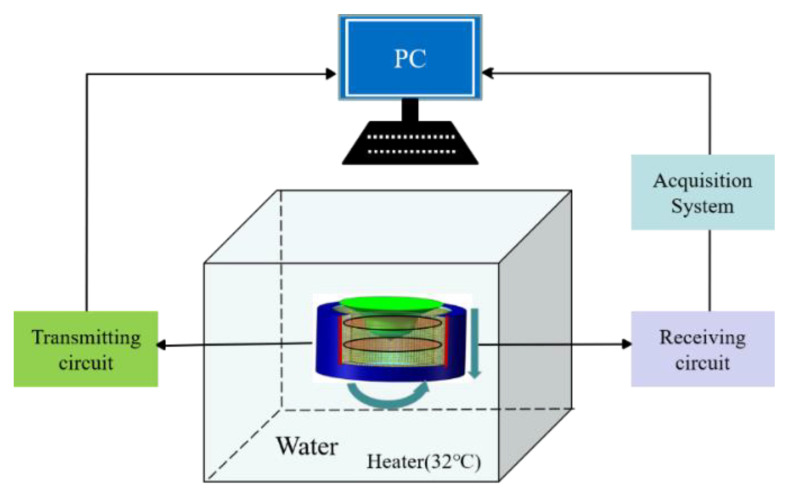
Ultrasonic tomography system based on a CMUT cylindrical array.

In this paper, a novel ultrasonic tomography system based on a CMUT ring array is designed (Figure 1) primarily for the detection of early breast cancer lesions.

**Figure 2:**
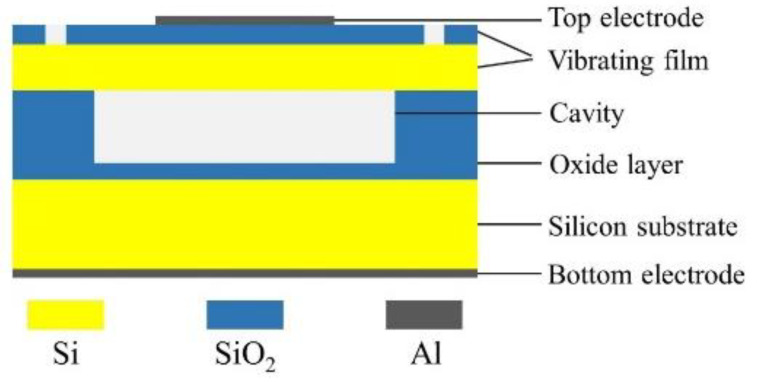
Structure of the CMUT cell.

**Figure 3:**
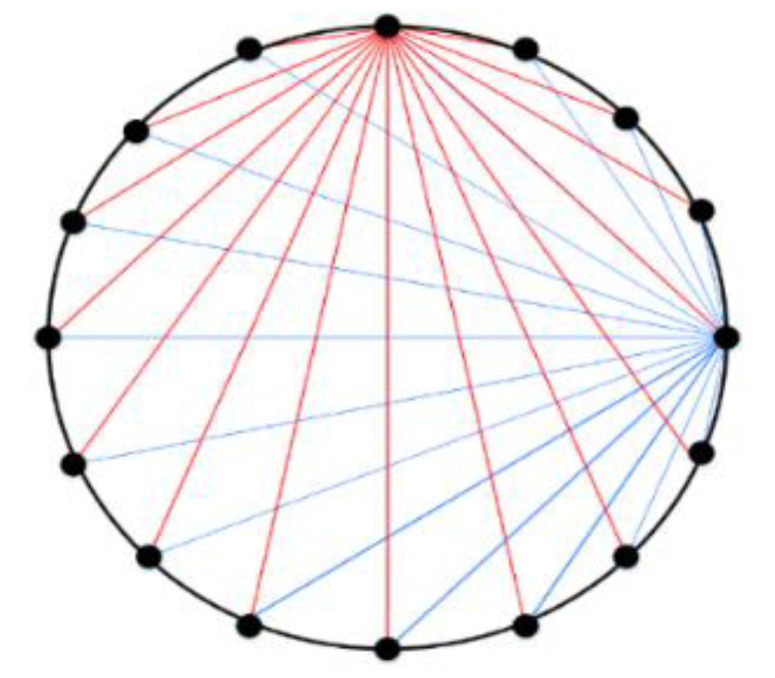
The scanning mode of ring array.

The system is mainly composed of a 1024-element cylindrical array detector, an ultrasonic signal transmitting and receiving control circuit, and a computer workstation. The structure of the CMUT cell is shown in Figure 2, which is mainly composed of a top electrode, a vibration film, a cavity, a oxide layer, a silicon substrate, and a bottom electrode. The diameter of the cell is 180 µm. Multiple cells are arranged in a honeycomb form to constitute a CMUT element. This system can perform multiple perspectives in ultrasound tomography by using the electronic scanning mode, which is shown in Figure 3. The one-transmission and full-receive acquisition mode is used to scan the breast model in 360°, that is, 1 to 1024 elements are used as a transmitting element to transmit ultrasonic signals successively, and all the elements receive ultrasonic signals at the same time.

## 3. Algorithm Formulation

A high computing efficiency and high-resolution reconstruction method for breast tissue acoustic characteristics is proposed, oriented to the CMUT ring array. This has both the advantages and disadvantages of existing acoustic parameter reconstruction algorithms and is required for accurate and rapid detection of small lesions in early breast cancer. Ultrasound propagation models based on wave theory are used to reconstruct sound speed, that is, the arrival time of ultrasound signals is recorded by all elements of the ring array. Ultrasound propagation models based on ray theory are used to reconstruct the attenuation coefficient, that is, the amplitude attenuation of ultrasound signals is recorded by the elements of the three-quarter ring array that are directly opposite to the transmitting element.

### 3.1. Sound Speed Inversion Algorithm

**Figure 4:**
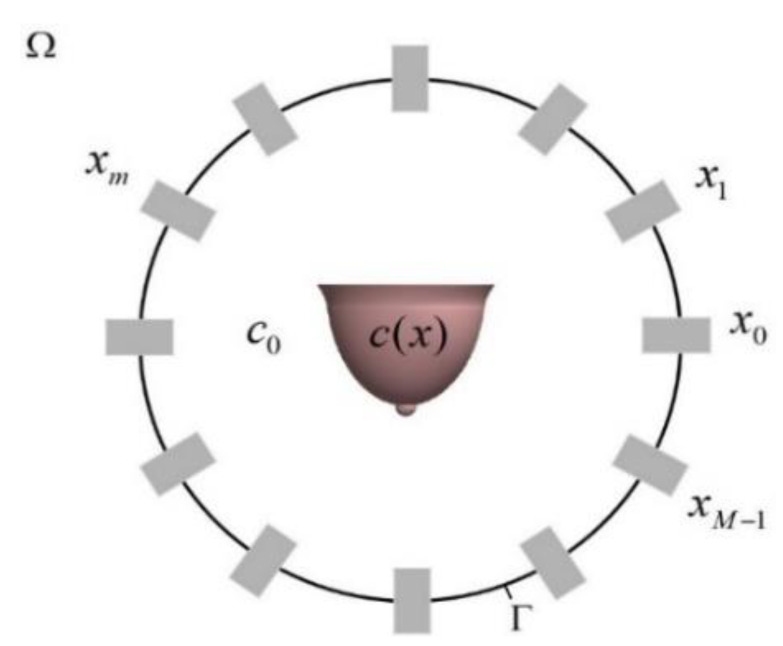
Sound speed ultrasound tomography setup.

Ultrasound transducer ring arrays composed of M elements distributed on the circular Γ at equal intervals are used as the transmitting and receiving devices, as shown in Figure 4.

The ring array surrounds the breast tissue for which the acoustic properties need to be imaged. In this part, the propagation speed of the ultrasound signal in breast tissue is estimated [2]. In the simulation domain Ω, it is assumed that sound propagation follows a two-dimensional wave equation:

(1) $$\nabla^{2}u(x,t)-\frac{1}{c^{2}(x)}\frac{\partial^{2}}{\partial t^{2}}u(x,t)=s(x,t)$$

where u(x,t) represents the sound pressure field at the position $x={\left(x_{1},x_{2}\right)}^{T}\in \Omega$, c(x) represents the ultrasound propagation speed in breast tissue, and s(x,t) represents the sound source signal. Generally, the m[th] transmitting transducer at the position $x_{m}$ is applied with a pulse signal to generate a wave field $u_{m}(x,t)$, for m=0,1,…,M−1, which is the solution to the wave in Equation (1). The propagation effect of sound source signal $S_{m}$ is expressed as a nonlinear operator ${ℜ}_{m}$, and it is defined as follows:

(2) $${ℜ}_{m}:f(x)\in L_{2}(\Omega )\mapsto g_{m}(r,t)\in L_{2}(\Gamma \times (0,T))$$

It maps the objective function f to the signal $g_{m}$ recorded on the circle Γ at the time interval (0,T). $L_{2}(\Omega )$ represents the set of square integrable functions over Ω. Thus, it holds that

(3) $${ℜ}_{m}(f)=g_{m},\;m=0,1,\ldots ,M-1$$

The goal of reconstruction algorithm is to solve the above nonlinear equations, that is, to recover the objective function f from the recorded signals $g_{m}$.

In order to solve the nonlinear Equation (3), the following cost functions need to be minimized:

(4) $$C(f)=\sum_{m=0}^{M-1}C_{m}(f)={\sum_{m=0}^{M-1}\left\|{ℜ}_{m}(f)-g_{m}\right\|}^{2}$$

With a linear relationship, the following update equation can be used to minimize the total cost function (4):

(5) $$f_{k}=f_{k-1}+\alpha \sum_{m=0}^{M-1}({ℜ}_{m}^{\prime }(f_{k}))*(g_{m}-{ℜ}_{m}(f_{k}))$$

where α is the step, which can be selected by line search method. The abovementioned updates depend on the calculation of two main variables. ${ℜ}_{m}(f)$ simply corresponds to the signal recorded on the circular Γ when sound source $s_{m}$ propagates at the sound speed given by the objective function f. For any $g_{\Gamma }\in L_{2}(\Gamma ,(0,T))$, the following relation holds:

(6) $$({ℜ}_{m}^{\prime }(f))*(g_{\Gamma })=\frac{1}{c_{0}^{2}}\int_{0}^{T}z_{m}\frac{\partial^{2}u_{m}}{\partial t^{2}}dt$$

where $z_{m}(x,t)$ is the solution to wave equation

(7) $$\nabla^{2}z_{m}(x,t)-\frac{1}{c^{2}(x)}\frac{\partial^{2}}{\partial t^{2}}z_{m}(x,t)=g(x,T-t)$$

When t<0, $z_{m}(x,t)=0$. Signal g(x,t) is a distribution defined by Equation (8)

(8) $$\int_{0}^{T}\int_{\Omega }g\varphi dxdt=\int_{0}^{T}\int_{\Gamma }g_{\Gamma }\varphi dxdt$$

where all $\varphi \in L_{2}(\Omega ,(0,T))$. The above equations mean that $\left({ℜ}_{m}^{\prime }(f))*(g_{\Gamma }\right)$ can be calculated from two different wave fields: (i) the field $u_{m}$ induced by source $s_{m}$ and (ii) a field $z_{m}$ emitted by a source g in time reversal. Apparent in the update (3), the source g is equal on the circle Γ to the residual between the measured signal $g_{m}$ and the signal ${ℜ}_{m}(f_{k})$ simulated using the current sound speed values. Algorithm 1 summarizes the reconstruction method of sound speed, as shown in Algorithm 1.

**Algorithm 1** Sound Speed Inversion1. $\mathrm{Start}\;\mathrm{with}\;\mathrm{an}\;\mathrm{initial}\;\mathrm{estimate}\;f_{0}$.2. For each source m,
Propagate the signal $s_{m}$ using the current estimate $f_{k}$ to obtain the field $u_{m}$,Compute the residual between the measured signal $g_{m}$ and the simulated signal ${ℜ}_{m}(f_{k})$,Propagate the residual in a time-reversed manner to obtain the field $z_{m}$.3. $\mathrm{Compute}\;\mathrm{the}\;\mathrm{update}\;f_{k+1}$ using (6) and (7).4. $\mathrm{If}\;\left|f_{k+1}-f_{k}\right|<\varepsilon$ for a prescribed threshold *ε*, stop. Otherwise, go to step 2.

### 3.2. Attenuation Coefficient Inversion Algorithm

**Figure 5:**
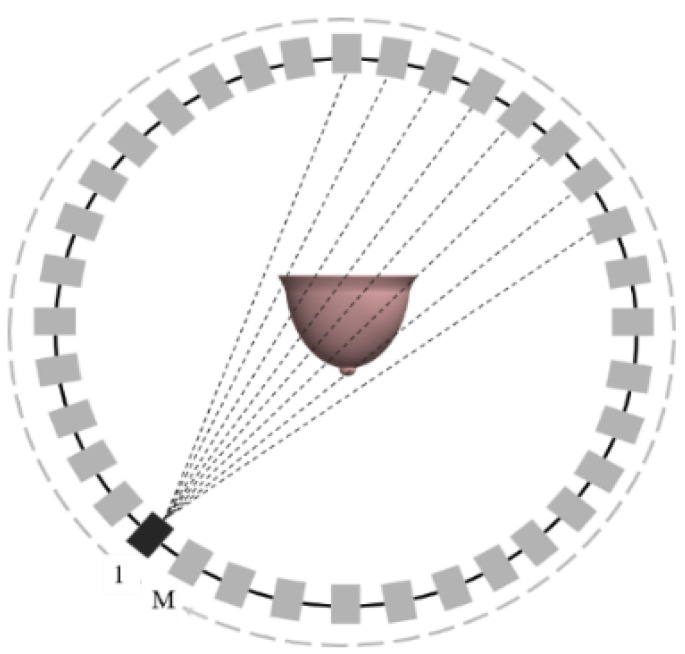
Attenuation coefficient ultrasound tomography setup.

Ultrasound transducer ring arrays composed of M elements distributed on the circular Γ at equal intervals were used as transmitting and receiving devices, as shown in Figure 5. A ring array surrounds the breast tissue for which the acoustic properties need to be imaged. In this part, the attenuation coefficient of the ultrasound signal in breast tissue is estimated [35,36].

**Figure 6:**
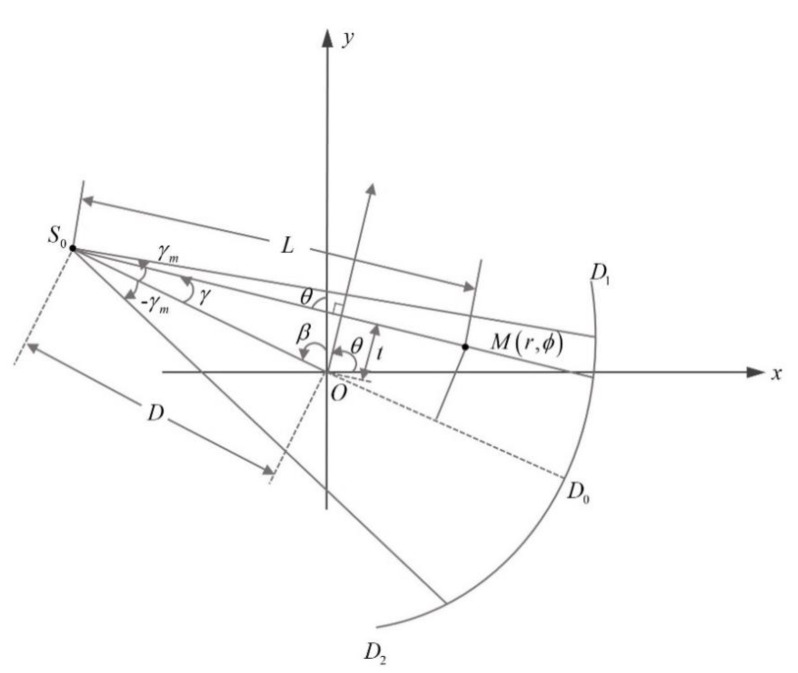
Geometric structure illustration of an equiangular fan beam system.

The attenuation coefficient of breast tissue was reconstructed by a filtered back-projection reconstruction method based on equiangular fan beam scanning. The parameter relationship of this method is shown in Figure 6, where the polar coordinates of point M in the reconstructed image are (r,ϕ), $S_{0}E$ is the ray passing through point M in fan beam, the flare angle is γ, and the length of line segment $S_{0}M$ is L. The relationships between the parameters satisfied Equation (9). p(t,θ) and $p^{f}\left(\gamma ,\beta \right)$ respectively represent the projections in a rotating coordinate system and in a polar coordinate system. t represents the distance from the origin of the rotating coordinate system to the ray.

(9) $$\begin{array}{l} \theta =\beta +\gamma \\ t=\mathrm{Dsin}\gamma \\ p(t,\theta )=p(D\sin\gamma ,\beta +\gamma )=p^{f}(\gamma ,\beta ) \end{array}$$

Therefore, the reconstruction formula of attenuation coefficient expressed by fan beam projection data and variables is as follows: h(γ) is a frequency domain filter.

(10) $$f(r,\phi )=\int_{0}^{2\pi }\frac{1}{L^{2}}\left[p^{f}(\gamma ,\beta )D\cos\gamma \right]*\frac{\gamma^{2}}{2\sin^{2}\gamma }h(\gamma )d\beta$$

where

(11) $$\begin{array}{l} L=\sqrt{D^{2}+r^{2}+2Dr\sin(\beta -\phi )}\\ \gamma =\arcsin\frac{r\cos(\phi -\beta )}{L}=\arcsin\frac{r\cos(\phi -\beta )}{\sqrt{D^{2}+r^{2}+2Dr\sin(\beta -\phi )}} \end{array}$$

Algorithm 2 summarizes the reconstruction method of attenuation coefficient, as shown in Algorithm 2.

**Algorithm 2** Attenuation Coefficient InversionInput: projection data (amplitude attenuation) $q\left(\gamma ,\beta \right)=p^{f}\left(\gamma ,\beta \right)$, angle β, angle of the interval between sampling rays γ.1. $\mathrm{Initial}\;\mathrm{data}\;\mathrm{processing}:\;\;q^{\prime }\left(\gamma ,\beta \right)=q\left(\gamma ,\beta \right)Dcos\gamma$.2. $\mathrm{Filter}\;\mathrm{function}\;h^{\prime \prime }(\gamma )$ to generate and convolute the projection data to obtain g(γ,β).3. Loop the coordinate of each pixel to be reconstructed in the main cycle, and loop from 0 to 360° in a sub-cycle, representing different angles between the central ray and the y axis. 4. $\mathrm{Calculate}\;\mathrm{the}\;\mathrm{angle}\;\gamma^{\prime }$ between the central ray and the line segment, connecting the point M  with the emission source, which corresponds to the central ray, calculated using the geometric relationship, and judge the positive and negative values corresponding to the central ray.5. $\mathrm{Divide}\;\gamma^{\prime }$ by γ to obtain the sampling index at this angle. If it is a decimal, it means that the point is in the middle of two projection rays and that interpolation was performed.6. Calculate the distance L between the pixels and the emission source.7. Interpolate linearly, and accumulate the projection data after filtering.
$Q=Q+(\gamma_{\theta }-floor(\gamma_{\theta }))\times g(floor(\gamma_{\theta }),\theta )+(ceil(\gamma_{\theta })-\gamma_{\theta })\times g(ceil(\gamma_{\theta }),\theta )/L^{2}$.8. End the sub-cycle and obtain the parameter value at that point through the cumulative sum.9. End the main cycle and obtain the parameter values of each point of the image through the cumulative sum.Output: Image of breast tissue attenuation coefficient.

## 4. Experimental Results

### 4.1. Numerical Experiments

**Figure 7:**
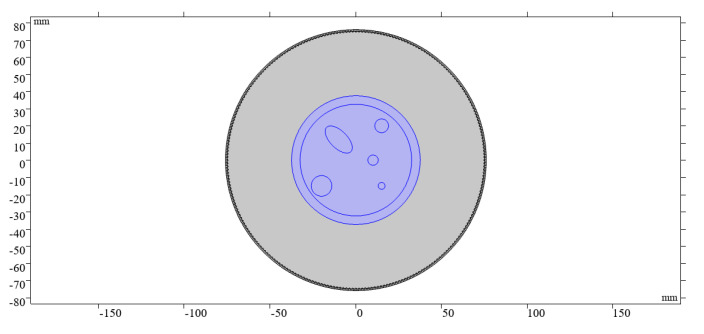
Ultrasonic imaging system model.

In order to verify the effectiveness of the reconstruction method proposed in this paper, projection data of ultrasound tomography imaging using the CMUT ring array are simulated by using the finite element simulation method. The ultrasound imaging system model shown in Figure 7 is established to obtain simulation data, which are close to the experimental data. The whole model is placed in an environment with water as a homogeneous background. The acquisition system is composed of a 1024-element ring array, which has a diameter of 14.8 cm, and it can completely surround the breast phantom, which includes the tumor, fibroma, cyst, calcification, fat, and glands. Specific parameters are shown in Table 1. The data acquisition process is as follows. One element is used to transmit the ultrasound signal, and all elements are simultaneously used to receive ultrasound signals each time. The 1024 elements are used as transmitting transducers in turn, and projection data are used for reconstruction of the sound speed. The attenuation coefficient can be obtained at the same time. In the whole circular scanning process, the projection data of 1024 angles were collected. The transmitted signal contained five cycles of the sine pulse signal for which the center frequency was 3 MHz and the value of peak to peak was 80 Vpp. Sound speed image updating was performed each time using a functional gradient estimated from the signals of 1024 receiving transducers. The attenuation coefficient image was reconstructed using the signals received by 768 receiving transducers directly opposite the transmitting transducer.

**Figure 8:**
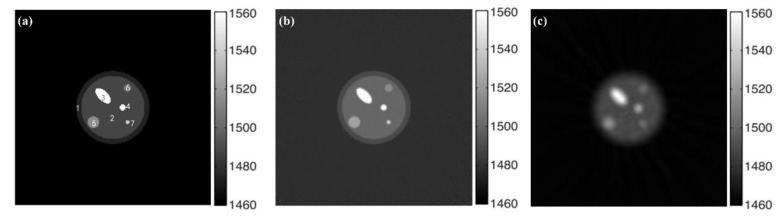
Reconstructed sound speed distribution. (**a**) Original sound speed distribution. (**b**) Reconstructed through time-reversal. (**c**) Reconstructed through FBP.

**Figure 9:**
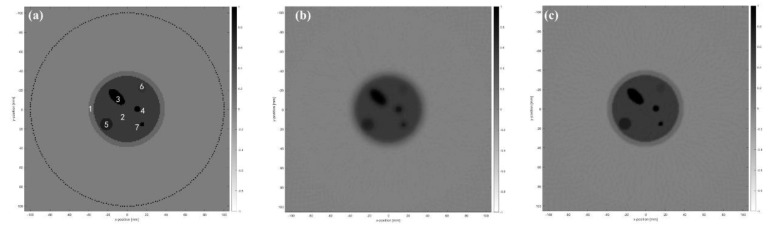
Reconstructed attenuation coefficient distribution. (**a**) Original attenuation coefficient distribution. (**b**) Reconstructed through time-reversal. (**c**) Reconstructed through FBP.

Reconstructed sound speed distributions and attenuation coefficient distributions are shown in Figure 8b,c and Figure 9b,c. In order to quantify the quality of the reconstruction, the average and standard deviation of pixel values in several ROIs located in the breast phantom are obtained and compared with the expected values of the acoustic characteristics (Table 2 and Table 3).

It can be seen from the reconstruction results of breast phantom that sound speed distribution can be recovered with higher precision through the time-reversal algorithm based on wave propagation theory than through FBP based on ray propagation theory. The attenuation coefficient distribution can be recovered with shorter calculation time and higher precision through FBP based on ray propagation theory than with a time-reversal algorithm based on wave propagation theory. According to the reconstruction result of sound speed (Figure 8 and Table 2), deviation between the reconstruction value and the expected value of all regions of interest are less than 0.16% using the time-reversal algorithm. Compared with the FBP algorithm, reconstruction bias is reduced by no less than 44%. Especially for the lesions for which the size is smaller than the wavelength, the reconstruction effect improved by 64.7%. According to the reconstruction result of the attenuation coefficient (Figure 9 and Table 3), deviation between the reconstruction value and the expected value of all regions of interest are less than 0.05% using the FBP algorithm. Compared with the time-reversal algorithm, the reconstruction deviation of its attenuation coefficient was reduced by 81.8%, especially for cysts and calcifications, which are difficult to detect in the early screening of breast cancer.

### 4.2. Breast Model Experiments

**Figure 10:**
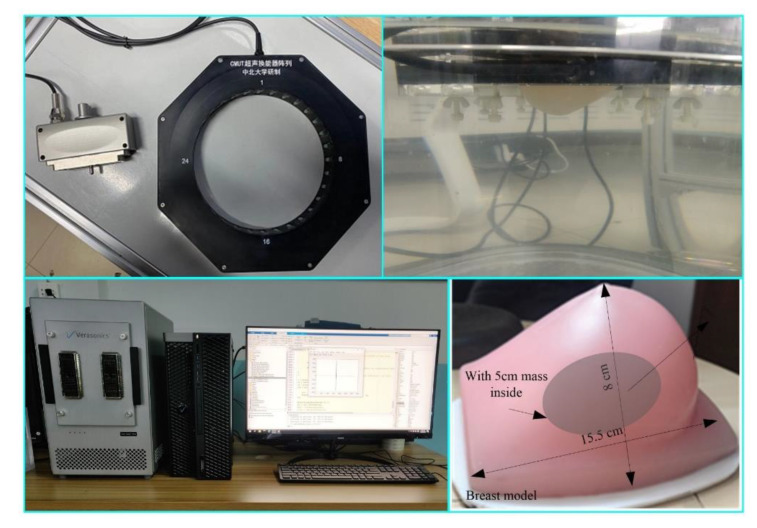
Experimental platform of breast ultrasound tomography system based on a CMUT ring array.

**Figure 11:**
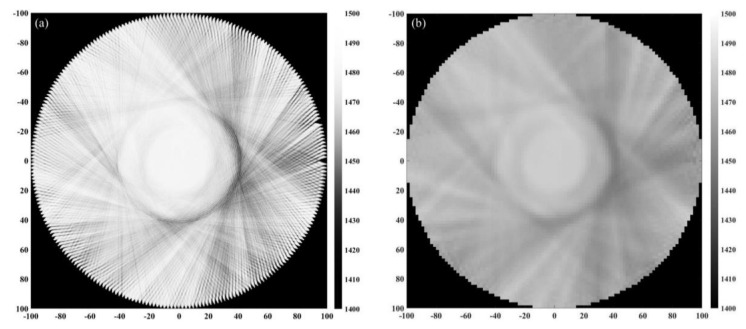
Reconstructed sound speed distribution. (**a**) Reconstructed through time-reversal. (**b**) Reconstructed through FBP.

**Figure 12:**
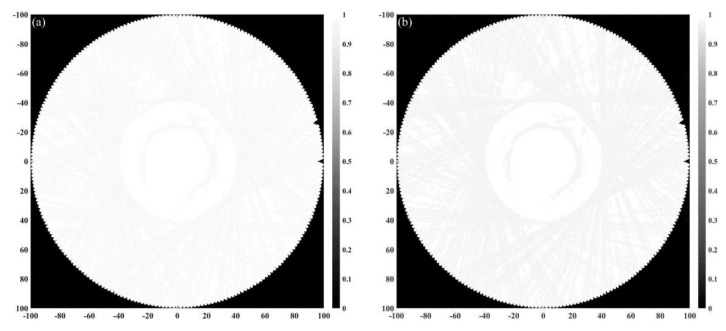
Reconstructed attenuation coefficient distribution. (**a**) Reconstructed through time-reversal. (**b**) Reconstructed through FBP.

In order to further verify the effectiveness of the reconstruction method proposed in this paper, the experimental platform shown in Figure 10 was built. The transducer used in this platform is a 32-element CMUT ring array with a diameter of 20 cm, in which the central frequency is 3 MHz. The diameter of the customized silicone breast model is shown in Figure 10, and its sound speed is about 1478 m/s, which is close to that of water. The inside of the breast model contains a mass with a diameter of 5 cm, and its sound speed is about 1491 m/s. The data acquisition method is the same as that in numerical simulation experiments. However, a circular scan can only obtain projection data from 32 angles, which cannot meet the data requirements of ultrasound tomography. Therefore, on the premise of the limited number of ultrasonic transducers, the 32-element CMUT ring array is rotated with an interval of 0.35° by using the electric rotary table a total of 32 times. Each time, 32 × 32 projection data are obtained, so a total of 1024 × 32 projection data can be obtained. Sound speed image updating is performed each time using a functional gradient estimated from the signals of 32 receiving transducers. The attenuation coefficient image is reconstructed using the signals received by 24 receiving transducers directly opposite the transmitting transducer. The reconstructed sound speed distribution and attenuation coefficient distribution are shown in Figure 11a,b and Figure 12a,b.

In order to further quantify the quality of reconstruction, the average and standard deviation of pixel values in mass located in the breast model are obtained and compared with the expected values of the acoustic characteristics (Table 4 and Table 5). According to the reconstruction results shown in Figure 11 and Table 4, the sound speed distribution reconstruction deviation can be reduced by 53.15% through a time-reversal algorithm based on wave propagation theory. Similarly, according to the reconstruction results shown in Figure 12 and Table 5, the attenuation coefficient distribution reconstruction deviation can be reduced by 61.53% through FBP based on ray propagation theory. The reconstruction effect is consistent with the simulation results, which verifies the feasibility of the reconstruction method proposed in this paper.

## 5. Summary and Conclusions

In this work, a high-precision, low time cost reconstruction method of breast tissue acoustic attenuation was proposed, in which the time-reversal reconstruction method and the ray acoustic method were used to reconstruct sound speed and acoustic attenuation, respectively. This method is designed for the application of the CMUT ring array in breast ultrasound computed tomography (CT) in order to realize rapid and high-resolution imaging of micro-lesions in early breast cancer, which has the advantages of high specificity and abundant information, because it is based on the propagation law of ultrasound in realistic breast tissue. First, the wave acoustic is the exact solution to a two-dimensional wave equation. Therefore, it can significantly improve the image resolution and realize accurate detection of small lesions by establishing an accurate ultrasound propagation to reconstruct the sound speed distribution of breast tissue. Second, acoustic attenuation is also an important acoustic characteristic to quantify structural characteristics of different breast tissues, which changes more with the tissue type and can provide enhanced contrast for different types of tissues. For these reasons, this paper focused on the reconstruction method of the breast attenuation coefficient. In addition, projection data required by the two methods can be obtained from the same received signals, which not only ensures the accuracy of image reconstruction but also improves the computational efficiency. It provides the possibility to accelerate the clinical application of breast USCT systems. In conclusion, the research results in this paper lay a good theoretical and practical foundation for the realization of a new generation of breast ultrasound tomography systems in the future.

## Figures and Tables

**Table 1:** Parameters of breast phantom.

ROI Number	Tissue Type	Size (mm)	Attenuation Coefficient (dB/MHz/cm)	Sound Speed (m/s)
1	Fat	r = 40	0.2	1470
2	Gland	r = 28	0.36	1480
3	Tumor	a = 15, b = 7.5	0.48	1560
4	Tumor	r = 3	0.48	1560
5	Fibroma	r = 6	0.21	1540
6	Cyst	r = 4.5	0.064	1510
7	Calcification	r = 1	0.5	1506

**Table 2:** Mean values, expected values, and bias at the ROIs shown in Figure 8 for sound speed distributions.

ROI Number	Mean Value (m/s)	FBP	Expected Value (m/s)	Bias (%)
		Time-Reversal		
1	1467.31		1470	0.18
	1471.34			0.10
2	1483.18		1480	0.21
	1481.47			0.10
3	1555.56		1560	0.35
	1557.72			0.15
4	1555.49		1560	0.33
	1558.26			0.13
5	1544.57		1540	0.29
	1541.21			0.078
6	1505.12		1510	0.34
	1511.89			0.12
7	1501.36		1506	0.29
	1504.49			0.10

**Table 3:** Mean values, expected values, and bias at the ROIs shown in Figure 9 for attenuation coefficient distributions.

ROI Number	Mean Value (m/s)	Time-Reversal	Expected Value (m/s)	Bias (%)
		FBP		
1	0.226		0.2	0.13
	0.194			0.03
2	0.335		0.36	0.07
	0.353			0.02
3	0.456		0.48	0.05
	0.478			0.004
4	0.516		0.48	0.071
	0.481			0.002
5	0.236		0.21	0.12
	0.217			0.03
6	0.037		0.064	0.42
	0.062			0.03
7	0.39		0.5	0.22
	0.48			0.04

**Table 4:** Mean value, expected value, and bias of the mass shown in Figure 11 for sound speed distribution.

ROI	Mean Value (m/s)	FBP	Expected Value (m/s)	Bias (%)
		Time-Reversal		
**Mass**	1474.5		1491	1.11
	1483.3			0.52

**Table 5:** Mean value, expected value, and bias of the mass shown in Figure 12 for attenuation coefficient distribution.

**ROI**	**Mean Value** **(m/s)**	**Time-Reversal**	**Expected Value** **(m/s)**	**Bias** **(%)**
		**FBP**		
**Mass**	0.081		0.094	13.83
	0.089			5.32
